# Richmond (Va.) Academy of Medicine and Surgery

**Published:** 1895-02

**Authors:** Mark W. Peyser

**Affiliations:** Secretary and Reporter


### Society Reports.

### RICHMOND (VA.) ACADEMY OF MEDICINE AND SURGERY.

JANUARY 8, 1895.

The officers for the year 1895 were installed at this meeting. Dr. Wm. J. Gordon, President; Dr. Mark W. Peyser, Secretary; Dr. Jno. F. Woodward, Treasurer.

Dr. HUGH M. TAYLOR read the paper for the evening: "Surgical Treatment of Spinal Traumatisms."

"It is necessary for us first to classify what we are going to treat and then determine how to treat it. The classification I shall make is: first, anatomical, as traumatisms, of the cord, membranes, bony canal and ligaments; second, clinically or pathologically, concussion (sic) of the spine, contusions, hemorrhages, inflammatory deposits, cicatricial contraction, and morbid changes incident to intra and extra-dural injuries, fractures, dislocations, and the train of symptoms following injuries to the nerves after they have left the cord. In order to apply intelligent treatment, it is necessary to differentiate between the manifestations of the different varieties of traumatism. It must be kept clear that all the manifestations to treat are results of pressure, as from effusions of lymph, serum or blood, clot, bony fragments, cicatricial contractions, etc.

According to most authorities, concussion of the spine is a misnomer, as the cord is so well protected by a water bath, by being anchored by the nerves in the bony cavity which is situated deep, lying in a mass of muscular tissue and held strongly together by ligaments. Injury while upright is rare, it occurring when the spine is flexed, as in the bending position. It is hard to realize that with these natural protections, there can be such a condition as concussion. It is really a part of shock. If the symptoms extend beyond the time in which shock should be recovered from, contusion is present.

Take into consideration the important part that the ligaments play in traumatisms of the spine: compare sprains of the spine with those of other parts and we must conclude that sprains of the spine are frequent. Many of the so-called injuries to the spine are ligamentous and not nervous. If a spinal ligament is torn or contused, it must be repaired by new tissue formation which may affect probably the nerves passing through, causing neuritic and trophic, motor or sensory disturbances. The damage may be outside and yet be manifested as above, *e.g.*, long continued neuralgia. Many of the so-called railway spines are simply such.

We do not often have the opportunity of studying contusions of the cord by *post-mortems*. We must study them by analogy, knowing all the results due to pressure.

The most common morbid condition is hemorrhage, the cord being richly supplied with blood vessels; and a contusion causing their rupture is not by any means rare.

It is important to differentiate pressure due to blood, serum or lymph. If the symptoms come on immediately, they are the result of fracture or dislocation; if in two or three hours, they are the result of pressure from hemorrhage; if in a day or two, serum is the cause; and if the symptoms of compression occur after weeks, they are due to lymph effusion.

The manifestations of pressure are: first, of the cord proper, depending on the part upon which pressure is exerted; second, of the spinal nerves, as interference with or destruction of their functions, as spasmodic contraction of the muscles supplied, paresis, paralysis, anæsthesia, hyperæsthesia, formication, etc. The symptoms are not only local, but remote; not only immediate, but subsequent.

It is not easy to say that the damage to a nerve is within or without the cavity. The reflex effects of nerve irritation are manifested at times when no plain explanation can be given. Mitchell and others say they are due to paresis of the higher centres.

TREATMENT.—The field of surgical treatment is limited, as when pressure from exudate exists. Traumatisms of the ligaments are not within surgical limits. Massage and extension should be employed for these. For damage to the cord, iodides, mercury, rest, douche, massage and moral treatment, which is important.

The only classes of injuries calling for surgical treatment are fractures and dislocations. Cut down on the spine to remove fragments and relieve pressure; but this is not an easy matter. It is advised to bite off the spinal and then the transverse processes until the cord is reached, and then be guided by indications as to the presence of clot or effusion, by the bulging, discoloration, etc.

It is conceded that the danger from hemorrhage or loss of spinal fluid is not as great as that of pressure.

Another method of treatment is absolute rest, extension to head and feet, and the use of sand-bags.

The bladder must be cared for and bed sores prevented. The reduction of fracture before operation is debatable, on account of the injury that may be inflicted in the process; and then it is doubtful if fracture or dislocation can be diagnosed without cutting down.

DR. MARK W. PEYSER reported the case of a man who, while mining, was struck on the back by a large mass of earth, resulting in dislocation of three vertebræ. Operation was of no benefit and the man succumbed. He also said that statistics show that tapping the cord for the relief of pressure was unavailing.

JANUARY 25, 1895.

At this meeting the President delivered his inaugural address: "Medical Societies; Their Aims, Benefits, Etc., Individual and Collective." It was well-prepared and filled with epigrams. "This is notably an age of organization." "The course of medicine is toward specialism, and this is engendering great competition. It is impossible for one mind to be proficient in all knowledge, but a man should at least be acquainted with various branches of the profession he has chosen." "Attrition of mind against mind, means benefit to both." "Union is strength, co-operation is strength with broadness of view, and fellowship is both and more."

REPORTS OF CASES.

DR. W. W. PARKER reported the case of a male, aged 48 years, who had been sick four or five years; and had been a valetudinarian for five or six years. There were no evidences of valvular trouble, but were of cardiac hypertrophy and some obscure liver trouble. During the last four or five months of his sickness, there had been dyspnœa and effusions into the pericardium. The patient suffered from insomnia, which nothing relieved until chloroform was used. *Post-mortem* revealed hypertrophied heart (no valvular lesions), effusion into the pericardium and evidences of supposed granulations of the liver. The doctor is awaiting the report of Dr. Hoge, pathologist, as to the latter.

DR. A. L. WELLFORD: Male, aged 36 years, robust, two months ago had been attacked with violent pains in the epigastrium radiating to both sides of the abdomen. These subsided in two or three days, but were followed by nausea, vomiting, extreme headache and pronounced bronchial rales. There were marked evidences of gastritis, and the vomited matter had a purulent appearance. Pressure on the right bronchus was discovered and rapid swallowing of large boluses of food showed stricture of the œsophagus. The kidneys were neuralgic; the urine showed an abnormal amount of phosphates, but otherwise, were negative. No heart murmur, cancer, syphilitic or strumous history. No aneurism. There was evidently a tumor pressing on the right bronchus, œsophagus, stomach, and probably, nerves, but its nature could not be discovered. The patient is thriving on bromide of sodium with hypophosphites of lime and sodium. Dr. James N. Ellis suggested that the pressure may have been due to an enlarged bronchial lymphatic gland in the mediastinum, but Dr. Wellford thought not.

DR. R. D. GARCIN wished to tell of a specimen of conservative surgery. Male, aged 76, had his right hand almost torn off by the band of an engine. Amputation was thought of, but the patient having heart trouble, was afraid to risk chloroformization and therefore stitched up the wound. In a couple of weeks a cast was put on, an opening in it being left for drainage. Bony union of the radius, but not of the ulna, occurred, and the man has now a useful hand.

DR. O. F. BLANKENSHIP: Female, aged 40, had been confined a week or two previous to his being called for a fainting spell which he thought merely syncope. The patient grew progressively worse, the spells came more frequently; she could not sit up in bed without having an attack, and palpitation was severe. Physical examination showed effusion into the pericardium.

At the time, the patient had no rheumatism, but had had it in the inflammatory form, previously. There was not much cardiac murmur. Autopsy revealed pericardial effusion and adhesion of the auriculo-ventricular valves to the heart walls. Length of time from first faint to death was one week.

Dr. J. F. CRANE: Girl, aged 10, seen at the Dispensary of the University College of Medicine. At varying intervals, the girl had *locked* jaw, the upper teeth being fixed in front of the lower, which are decayed. Unlocking is an easy process. The doctor thinks the trouble is due to relaxation of the temporo-maxillary ligaments and dislocation of the interarticular fibro-cartilage. Dr. Michaux doesn't think Dr. Crane's diagnosis is clear. In cases of this kind the jaw is locked; but open, not closed. He is of the opinion that the trouble is muscular and probably reflex. Dr. J. W. Henson, who has seen the case, thinks Dr. Crane is right, and that Dr. Michaux's remarks apply to dislocation, of which this is not a case.

DR. J. W. HENSON cited the case of an infant of 8 weeks, who had umbilical hernia with no covering whatever, the intestines protruding through a hole in the abdominal wall and lying on it. It occurred in 12 hours and was as large as his two fists. The child died in 24 hours of peritonitis. The doctor has never seen or heard of a similar case.

DR. JAMES N. ELLIS reported the case of a boy who had had Pott's disease. Pieces of necrosed bone gained access to the abdominal cavity, perforating its wall at the umbilicus. Autopsy revealed pieces of bone floating in pus within the cavity.

MARK W. PEYSER, M. D.,
Secretary and Reporter.

